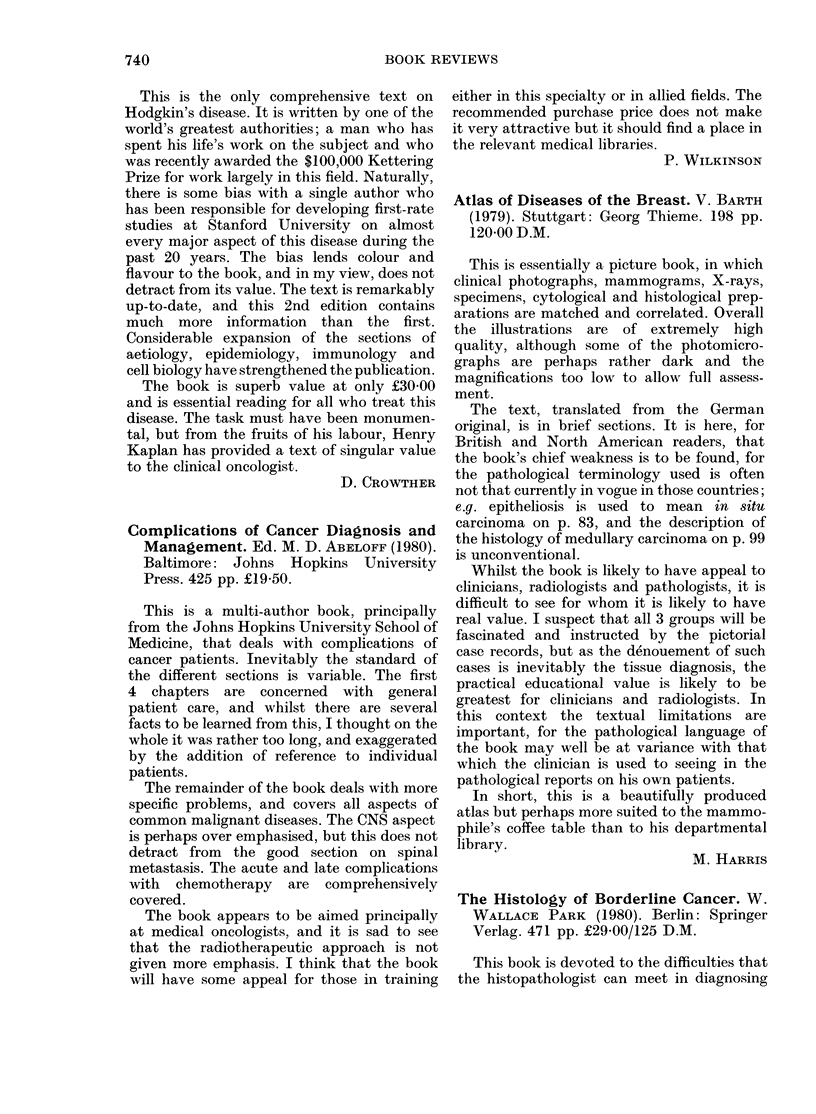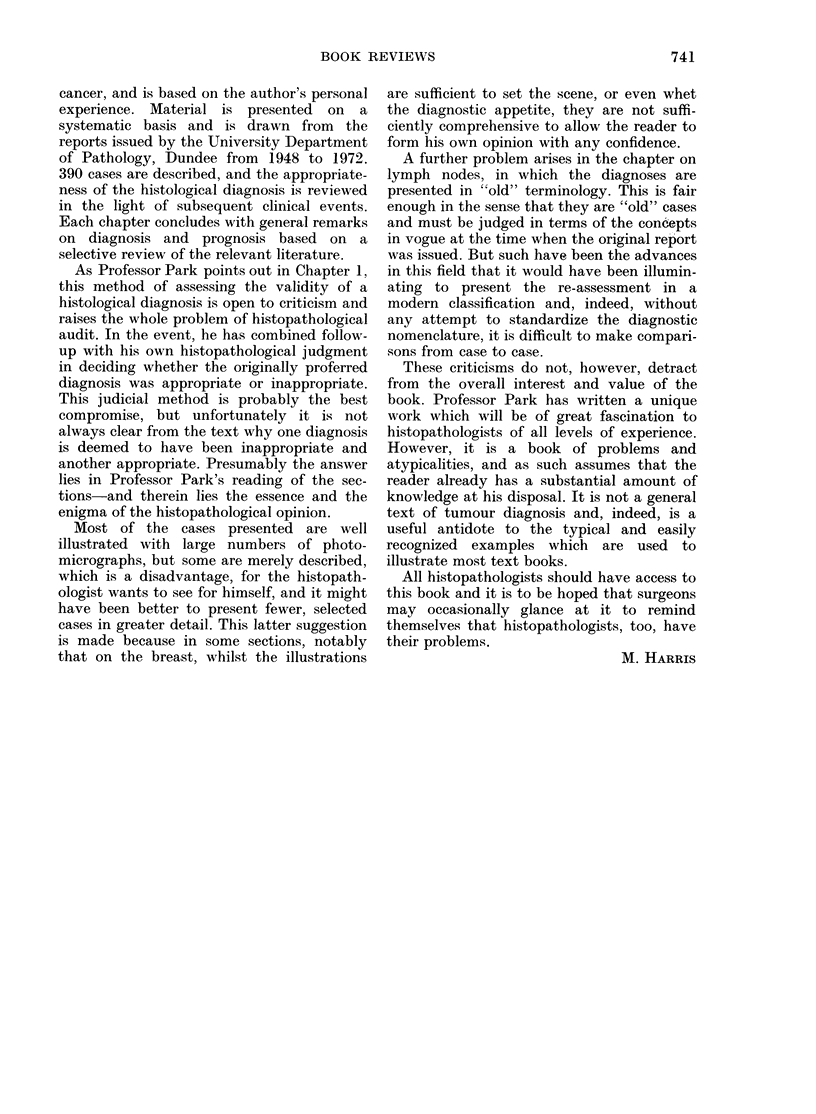# The Histology of Borderline Cancer

**Published:** 1981-05

**Authors:** M. Harris


M. Harris

**The Histology of Borderline Cancer.** W. Wallace Park (1980). Berlin: Springer Verlag. 471 pp. £29·00/125 D.M.

This book is devoted to the difficulties that the histopathologist can meet in diagnosing cancer, and is based on the author's personal experience. Material is presented on a systematic basis and is drawn from the reports issued by the University Department of Pathology, Dundee from 1948 to 1972. 390 cases are described, and the appropriateness of the histological diagnosis is reviewed in the light of subsequent clinical events. Each chapter concludes with general remarks on diagnosis and prognosis based on a selective review of the relevant literature.

As Professor Park points out in Chapter 1, this method of assessing the validity of a histological diagnosis is open to criticism and raises the whole problem of histopathological audit. In the event, he has combined follow-up with his own histopathological judgment in deciding whether the originally proferred diagnosis was appropriate or inappropriate. This judicial method is probably the best compromise, but unfortunately it is not always clear from the text why one diagnosis is deemed to have been inappropriate and another appropriate. Presumably the answer lies in Professor Park's reading of the sections—and therein lies the essence and the enigma of the histopathological opinion.

Most of the cases presented are well illustrated with large numbers of photomicrographs, but some are merely described, which is a disadvantage, for the histopathologist wants to see for himself, and it might have been better to present fewer, selected cases in greater detail. This latter suggestion is made because in some sections, notably that on the breast, whilst the illustrations are sufficient to set the scene, or even whet the diagnostic appetite, they are not sufficiently comprehensive to allow the reader to form his own opinion with any confidence.

A further problem arises in the chapter on lymph nodes, in which the diagnoses are presented in "old" terminology. This is fair enough in the sense that they are "old" cases and must be judged in terms of the concepts in vogue at the time when the original report was issued. But such have been the advances in this field that it would have been illuminating to present the re-assessment in a modern classification and, indeed, without any attempt to standardize the diagnostic nomenclature, it is difficult to make comparisons from case to case.

These criticisms do not, however, detract from the overall interest and value of the book. Professor Park has written a unique work which will be of great fascination to histopathologists of all levels of experience. However, it is a book of problems and atypicalities, and as such assumes that the reader already has a substantial amount of knowledge at his disposal. It is not a general text of tumour diagnosis and, indeed, is a useful antidote to the typical and easily recognized examples which are used to illustrate most text books.

All histopathologists should have access to this book and it is to be hoped that surgeons may occasionally glance at it to remind themselves that histopathologists, too, have their problems.

M. Harris